# Of Mice and Men: Empirical Support for the Population-Based Social Epistasis Amplification Model (a Comment on Kalbassi et al., 2017)

**DOI:** 10.1523/ENEURO.0280-17.2017

**Published:** 2017-09-21

**Authors:** Matthew Alexandar Sarraf, Michael Anthony Woodley of Menie

**Affiliations:** 1 University of Rochester, Rochester, NY 14627; 2 Center Leo Apostel for Interdisciplinary Studies, Vrije Universiteit Brussel, Brussels B-1160, Belgium; 3 Unz Foundation, Palo Alto, CA

**Keywords:** depression, fitness, group selection, mutation accumulation, social epistasis, social hierarchy

## Abstract

This commentary article offers new perspective on recent research investigating the behavioral and social ecological effects of a mutation related to autism spectrum disorders in mice. The authors explain the consistency of this research on mice with predictions advanced by a theory of the role of mutations in altering interorganismal gene-gene interactions (social epistasis) in social species including humans, known as the social epistasis amplification model. The potential significance of the mouse research for understanding contemporary human behavioral trends is explored.

## Significance Statement

The authors explain the consistency of Kalbassi et al. (2017)'s research on mice with predictions advanced by a theory of the role of mutations in altering interorganismal gene-gene interactions (social epistasis) in social species, known as the social epistasis amplification model. In light of this model, the mouse research has implications for understanding some of the most important contemporary human behavioral trends.

## 

Kalbassi et al. (2017) examined whether and how mice carrying a behavior-altering mutation change their social ecologies, which is to say their behavior and that of their conspecifics. Specifically, the authors investigated the effect of deletion of the gene *Nlgn3*, which is related to autism spectrum disorders, on the phenotypes of both “*Nlgn3* knockout mice” and their “wild-type littermates” (i.e., mice without the deletion of *Nlgn3*) with which they were raised (p. 1). The authors determined that litters containing male mice both with (*Nlgn3^y/-^*) and without (*Nlgn3^y/+^*) deletion of *Nlgn3* were substantially different from litters containing only *Nlgn3^y/+^* mice (the authors also examined litters containing only *Nlgn3^y/-^*mice and various litters of female mice, but this commentary does not focus on these cases). Among the more striking findings are that the genotypically mixed compared to homogeneous litters lacked “a structured social hierarchy” (p. 9) and had lower levels of testosterone (in both *Nlgn3^y/-^* and *Nlgn3^y/+^*mice); additionally, *Nlgn3^y/+^*mice from genotypically homogeneous litters showed more interest in “social” as opposed to “non-social cues” (p. 9) than *Nlgn3^y/+^*mice from genotypically mixed litters [the latter did not show a preference for one type of cue over the other, “showing an absence of interest for social cues” (p. 9)]. However, “re-expression of *Nlgn3* in parvalbumin-expressing interneurons in *Nlgn3^y/-^* mice rescues their social submission phenotype and the corresponding effect on the wild-type littermates” (p. 2). The authors infer that their findings, taken collectively, indicate not only an effect of deletion of *Nlgn3* on the phenotypes of mice carrying the mutation, but also on *Nlgn3^y/+^*mice with which the carriers were raised. [Kalbassi et al. (2017) note that it appears not only that *Nlgn3^y/-^*mice affect the phenotypes of *Nlgn3^y/+^*mice, but that the latter affect the phenotypes of the former.]

These results should be considered in the context of the broader body of theoretical and empirical work with which they are consistent. Most saliently, a paper published earlier this year integrated a great deal of research on social epistasis, i.e., interorganismal gene-gene interactions, and mutation load in humans, mice, and other organisms to develop the novel thesis that the fitness costs of the accumulation of certain kinds of deleterious mutations under conditions of relaxed negative selection in humans are externalized onto noncarrier individuals and thereby amplified via the damage that these mutations do to populations’ “group-level extended phenotype[s]” (Woodley of Menie et al., 2017). This theory, which was termed the social epistasis amplification model, is based partly on the biological literature concerning eusocial insects, in which the term “social epistasis” was coined (e.g., Linksvayer, 2007). Research on such insects has found that the adaptively optimal development of members of different castes in insect societies depends on certain intercaste genotypic interactions, or social epistases. Thus both the individual- and group-level fitness of eusocial insects, insofar as both are contingent on (inter- and intra-)caste structure and cooperation, depend on the existence of particular social distributions of genotypes and epistatic interactions among them. This fact suggests the possibility that, as a general rule, social species, including humans, require certain patterns of interorganismal genetic interactions to achieve and maintain adaptive optima at the individual and group levels (see Domingue & Belsky, 2017 for a detailed overview).

Just as the *Nlgn3^y/-^* mice damaged their immediate community ecology, e.g., by inhibiting the development of normal social hierarchies and driving down the testosterone levels of their *wild-type* litter-mates, because they carried a mutation, it is possible that the carriers of behavior-altering mutations in contemporary human populations reduce the fitness of their noncarrier counterparts and depress group-level fitness overall. Deletion of *Nlgn3* is potentially a “spiteful mutation” (Woodley of Menie et al., 2017). A mutation is spiteful if it degrades the fitness of carriers and also undermines, or incurs opportunity costs on, the fitness of conspecifics with whom carriers enter into social epistatic transaction, e.g., by imposing sociocultural conditions that disincentivize procreation. Woodley of Menie et al. (2017) ran structured population models to explore the effects of spiteful mutations, which found that under relaxed negative selection, these mutations reach fixation because population growth offsets their individual-level fitness costs. But once a critical prevalence of mutations is present, their negative impact on the fitness of noncarriers causes rapid population decline. With negative selection against the carriers of these spiteful mutations restored, population growth continues until a stable equilibrium is established. Thus, Kalbassi et al. (2017) found, as Woodley of Menie et al. (2017)’s model predicts, that restoration of adaptive behavior occurred in mouse communities when *Nlgn3* was re-expressed in *Nlgn3^y/-^*mice (this restoration simulates the effects of negative selection as reflected in Woodley of Menie et al., 2017’s model).

Moreover, there is much empirical evidence that a number of human populations are experiencing increasing loads of behavior-altering, and fitness-reducing, mutations. In at least some Western populations, the prevalence of several mental disorders associated with advanced paternal age [because older fathers bequeath more mutations to their offspring than younger ones (Rahbari et al., 2016)], most notably unipolar depression (Laursen et al., 2007) and autism (Kong et al., 2012), has substantially increased from the 20th to 21st centuries (Twenge et al., 2010; Russell et al., 2015). Furthermore, schizophrenia, another disorder associated with paternally acquired *de novo* mutations (Malaspina et al., 2002), may have been extremely rare before AD 1800 (Hare, 1988), but became more common thereafter, possibly because 19th century industrialization lowered the fitness costs associated with the disease. These secular trends, and others suggesting rising rates of subclinical behavioral abnormalities also (e.g., Greenfeld, 2013, pp. 621–622), could indicate a growing prevalence of spiteful mutations in particular populations. Additionally, Kalbassi et al. (2017)’s linking of the presence of *Nlgn3^y/-^*mice to testosterone decline across the board in the latter’s communities yields a compelling potential explanation for the observation of significantly declining testosterone levels in Western males (Travison et al., 2007). In the same demographic, sperm quality has been diminishing precipitously for at least the past few decades (Levine et al., 2017), a phenomenon in which the aforementioned losses of testosterone may be implicated. Certain cultural changes, such as secularization, have also been connected to fertility decline at the group and individual levels (Meisenberg, 2011), and there is evidence that irreligiosity is associated with behavioral and physical abnormalities indicative of higher relative burdens of deleterious mutations (Dutton, 2017). Religion is a group-level adaptation in humans (Wilson, 2002), as is social hierarchy in mice (van den Berg et al., 2015). Thus, the carriers of spiteful mutations may disrupt the patterns of social epistasis that sustain religiosity and other group-level adaptations, and consequently lower fitness.

Woodley of Menie et al. (2017) predicted that results similar to Kalbassi et al. (2017)’s would be found in mice subjected to the proper experimental design. Kalbassi et al. (2017) are to be commended for devising a better procedure than that contained in the provisional experiment offered by Woodley of Menie et al. (2017)—which is a variant of the “mouse utopia” experiments of Calhoun (1973)—wherein two mice colonies were to be bred under cornucopian conditions. The colony serving as a control would be an effective replication of Calhoun’s own mouse utopia, where, like Calhoun’s colony, the mouse population would presumably cycle through to collapse. However, in the case of the experimental colony, lines showing signs of having accumulated behavior-altering mutations would be treated with *CRISPR* (a gene-editing technology) to remove such mutations. If the experimental colony could be indefinitely sustained and avoid the population collapse of the control colony, this would strongly evidence that deleterious mutation accumulation, permitted by conditions of minimized morbidity and mortality, caused the control colony’s breakdown. Since Kalbassi et al. (2017) observed that adaptive behavior was restored in communities with both *Nlgn3^y/-^* and *Nlgn3^y/+^*, and that restoration occurred in both types of mice, when *Nlgn3* was re-expressed in *Nlgn3^y/-^*mice, they have effectively demonstrated what Woodley of Menie et al. (2017)’s proposed experiment was predicted to show. It is also interesting to note that Calhoun (1973) observed that the decline of the mouse utopia colonies was accompanied by an increase in the prevalence of behaviorally abnormal mice—termed “beautiful ones.” Calhoun described these mice as “autistic-like creatures” (Calhoun, 1973, p. 86)—might these mice have possessed genotypes similar to the knockout mice examined by Kalbassi et al. (2017)?

Finally, a potential extension of the experiments of Kalbassi et al. (2017) would be to set up competition over limited resources (such as restricted access to food, territory, etc.) between differentially tagged groups of male mice [one group genotypically heterogeneous (containing *Nlgn3^y/+^*and *Nlgn3^y/-^* mice) and the other homogeneous (containing only *Nlgn3^y/+^*mice)]. Given the suppressing effect of the presence of *Nlgn3^y/-^* mice on the testosterone levels of their *wild-type* littermates, and given the hierarchy-avoidant behaviors that seemingly result, it is predicted that the homogeneous colony would outcompete the heterogeneous one, indicating greater group-level fitness. Competing differentially tagged groups of mice that are composed only of *Nlgn3^y/+^*mice could serve as a control for such an experiment, as the outcome of the latter competition in terms of which group gains the upper-hand should be due to contingent factors and would therefore essentially be random.

## Synthesis

Reviewing Editor: Carmen Sandi, Swiss Federal Institute of Technology Decisions are customarily a result of the Reviewing Editor and the peer reviewers coming together and discussing their recommendations until a consensus is reached. When revisions are invited, a fact-based synthesis statement explaining their decision and outlining what is needed to prepare a revision will be listed below.

This commentary article offers a new perspective on the recently published article by Kalbassi et al. (2017) in eNeuro. Findings in the original article are authoritatively put into the context of previous work and predictions by the theory of the role of interorganismal gene-gene interactions. In addition, the commentary emphasizes the translational relevance of the original work in mice to understand similar phenomena in humans.
